# A combination of HPLC and automated data analysis for monitoring the efficiency of high-pressure homogenization

**DOI:** 10.1186/s12934-017-0749-y

**Published:** 2017-08-01

**Authors:** Britta Eggenreich, Vignesh Rajamanickam, David Johannes Wurm, Jens Fricke, Christoph Herwig, Oliver Spadiut

**Affiliations:** 1 Research Division Biochemical Engineering, Institute of Chemical, Environmental and Biological Engineering, TU Wien, Gumpendorfer Strasse 1a, 1060 Vienna, Austria; 2 Christian Doppler Laboratory for Mechanistic and Physiological Methods for Improved Bioprocesses, Institute of Chemical, Environmental and Biological Engineering, TU Wien, Gumpendorferstraße 1a, 1060 Vienna, Austria

**Keywords:** Cell disruption, High-pressure homogenization, *E.coli*, HPLC, Data analysis

## Abstract

**Background:**

Cell disruption is a key unit operation to make valuable, intracellular target products accessible for further downstream unit operations. Independent of the applied cell disruption method, each cell disruption process must be evaluated with respect to disruption efficiency and potential product loss. Current state-of-the-art methods, like measuring the total amount of released protein and plating-out assays, are usually time-delayed and involve manual intervention making them error-prone. An automated method to monitor cell disruption efficiency at-line is not available to date.

**Results:**

In the current study we implemented a methodology, which we had originally developed to monitor *E. coli* cell integrity during bioreactor cultivations, to automatically monitor and evaluate cell disruption of a recombinant *E. coli* strain by high-pressure homogenization. We compared our tool with a library of state-of-the-art methods, analyzed the effect of freezing the biomass before high-pressure homogenization and finally investigated this unit operation in more detail by a multivariate approach.

**Conclusion:**

A combination of HPLC and automated data analysis describes a valuable, novel tool to monitor and evaluate cell disruption processes. Our methodology, which can be used both in upstream (USP) and downstream processing (DSP), describes a valuable tool to evaluate cell disruption processes as it can be implemented at-line, gives results within minutes after sampling and does not need manual intervention.

## Background

**Fig. 1:**
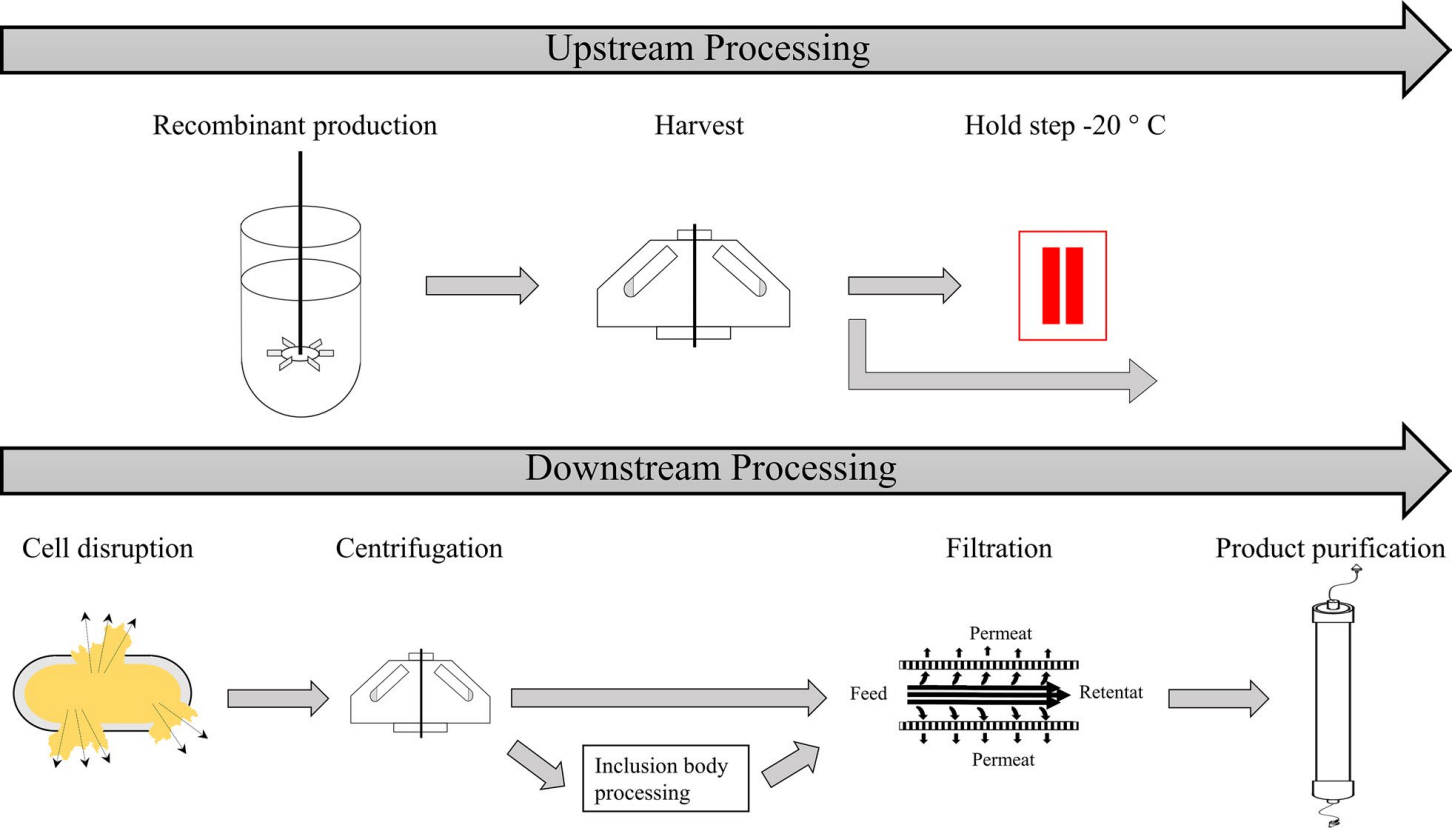
Typical recombinant protein production process in *E. coli*. After harvest, the biomass can optionally be frozen (indicated in *red*) before cells are disrupted and recombinant product is purified

Biopharmaceuticals represent the most valuable product class on the pharmaceutical market today [1]. The first recombinant biopharmaceutical, a human insulin analogue, was produced in *E. coli* and introduced to the market already in 1982 [1, 2]. Since then, *E. coli* has become one of the most important host organisms for the recombinant production of biopharmaceuticals. Currently, more than 25% of approved biopharmaceuticals are expressed in this organism [3]. This can easily be explained as *E. coli* allows fast growth in defined media and gives high product titers in scalable processes, resulting in economic production processes [1, 2, 4]. However, *E. coli* cannot perform post-translational modifications, limiting the product range that can be produced in a soluble and active form in this host organism [5]. Furthermore, *E. coli* cannot secrete recombinant proteins. Consequently, recombinant *E. coli* cells need to be disrupted to access the intracellular product, which is then usually purified by several steps of filtration and chromatography [5–7]. A typical recombinant *E. coli* protein production process is schematically shown in Fig. 1.

As shown in Fig. 1, the downstream process usually starts with the unit operation “Cell disruption”. There is a variety of different methods available to disrupt *E. coli* cells and make the intracellular product accessible. In Table 1, the most common principles and methods for cell disruption as well as the respective advantages and disadvantages are summarized.

**Table 1:** Most common cell disruption principles, respective methods as well as advantages and disadvantages

Principle of cell disruption	Method	Advantages	Disadvantages	References
Chemical	Detergents, solvents, acid, base	Standard lab equipment sufficient, selective release	Expensive, not scalable, not controllable	[8–11]
Biological	Lysozyme	Standard lab equipment sufficient	Expensive, not scalable, additional impurity	[12, 13]
Physical	Freeze–thawing	Standard lab equipment sufficient	Not scalable, inefficient	[14]
	Acoustic cavitation	Fast, efficient, easy handling	Not scalable, time consuming	[12, 14, 15]
	Hydrodynamic cavitation	Selective release	Inefficient, not scalable	[10, 16, 17]
	Osmotic shock	Selective release	Time consuming, not scalable	[10, 11, 18]
Mechanical	Grinding (e.g. bead mill)	Efficient	Time consuming, not scalable, generation of heat	[10, 11, 14, 15]
	High-pressure homogenization	Efficient, scalable	Generation of heat	[13, 15, 18–21]

Independent of the applied cell disruption principle and the respective method, each cell disruption process must be evaluated with respect to disruption efficiency and potential product loss. On the one hand, cell disruption must be efficient to obtain the maximum of intracellular product. On the other hand, however, excessive treatment of the cells might negatively affect the highly valuable product. The most common methods to evaluate cell disruption efficiency and their respective advantages and disadvantages are summarized in Table 2.

**Table 2:** Common methods to evaluate cell disruption efficiency

Answer	Method	Advantage	Disadvantage	References
Total protein release	Protein concentration (e.g. Bradford, Lowry)	Relatively fast, standard lab equipment sufficient	Matrix interference, manual intervention	[8, 15, 18, 21]
Cell viability	Microscope/flow cytometry	Detailed information	Error prone, dyes needed, expensive	[20, 21]
	Plate out (Colony forming Units (CfUs))	Standard lab equipment sufficient	Error prone, time consuming, laborious	[18, 20, 22, 23]
Product specific assays	SDS-Page, Western blot, ELISA, enzyme assays	Product specific	Time consuming, laborious, manual intervention	[12, 13, 15, 24]
Particle size distribution	Light scattering (e.g. Coulter Multisizer II, Nanophox PCCS)	Detailed information	Manual intervention, time consuming	[13, 18, 19, 24]

As shown in Table 2, the current state-of-the-art methods to evaluate cell disruption efficiency are usually offline and time-consuming and often need manual intervention making them highly error-prone. Thus, there is a demand for a methodology that allows automated evaluation of cell disruption efficiency without great time delay. Only by such a tool unnecessary cell disruption cycles in manufacturing can be avoided and therefore product loss minimized.

In the present study, we demonstrate how an HPLC method in combination with automated data analysis, which we originally developed for monitoring *E. coli* cell integrity during bioreactor cultivations [25], can solve the current issues in evaluating cell disruption efficiency. We compared different methods to evaluate cell disruption efficiency by high-pressure homogenization (Table 1) and proved the applicability of our automated method, which will definitely facilitate and accelerate bioprocess development in the future, since it describes a powerful tool applicable across unit operations. We did not investigate the effects of cell disruption strategies on product loss, since this is highly product-specific and thus must be evaluated on a case-by-case basis, but rather provide a platform tool to automatically evaluate cell disruption efficiency at-line.

## Materials and methods

### Chemicals

All chemicals were purchased from Carl Roth GmbH (Vienna, Austria), if not stated otherwise.

### Strains and cultivations

#### Strain

All experiments were performed with a recombinant *E.coli* BL21(DE3) strain producing a recombinant single chain fragment variable (scFv) against gliadin, which causes coeliac disease [26].

#### Shake flask cultivations

A 500 mL shake flask (SF) containing 50 mL sterile Super Broth medium (tryptone 32 g/L, yeast extract 20 g/L, NaCl 5 g/L, pH 7.2 ± 0.2) supplemented with 50 µg/mL kanamycin (SB-Kan) was inoculated from a frozen stock (1.5 mL, −80 °C). This pre-culture was incubated at 37 °C and 230 rpm in an Infors HR Multitron shaker (Infors, Bottmingen, Switzerland) for 12 h. Then, 490 mL sterile SB-Kan in a 2500 mL ultra-high-yield SF were inoculated with 10 mL pre-culture. The main culture was incubated at 37 °C and 230 rpm until the optical density at 600 nm (OD_600_) reached between 0.5 and 0.7. Then the culture was induced with 1 mM isopropyl β-d-1-thiogalactopyranoside (IPTG) for 16 h. To estimate the dry cell weight (DCW) of the cultivation broth an already generated OD_600_-DCW/L correlation was used (Eq. 1),

1 $$y = 0.451 \cdot x,$$

where x represents the measured OD_600_ value and y the DCW/L cultivation broth. Thus, aliquots with predefined biomass (BM) concentrations for subsequent homogenization were prepared.

#### Bioreactor cultivations

Bioreactor cultivations were performed according to our previous study [27]. In short, 500 mL pre-culture (DeLisa medium [28]; 50 µg/mL kanamycin) were used to inoculate 4500 mL sterile DeLisa medium in a stainless steel Sartorius Biostat Cplus bioreactor (Sartorius, Göttingen, Germany) with a working volume of 10 L. After a batch and a non-induced fed-batch, cells were induced by 1 mM IPTG at 30 °C for 8 h.

### Harvest and cell disruption

The cultivation broth was aliquoted and cells were harvested by centrifugation (4500 rpm, 4 °C, 30 min). Supernatants were discarded and cell aliquots were either frozen at −20 °C, representing a potential holding step in the process (Fig. 1), or processed immediately. Prior to cell disruption, frozen or fresh biomass (BM) pellets were resuspended in 50 mM TRIS–HCl buffer, pH 8.0. Cell suspensions were adjusted to 10 g DCW/L, if not stated otherwise.

In the present study, we performed cell disruption by high-pressure homogenization using a PandaPLUS 2000 (GEA Mechanical Equipment, Parma; Italia). At first, resuspended BM was pumped in cycles through the homogenizer to remove residual air. After applying the pre-pressure, the main-pressure was adjusted to 1500 bar to disrupt the cells, if not stated otherwise. To limit heat generation, BM was kept on ice and a cooling unit was connected to the outlet of the homogenizer. After resuspending biomass in TRIS–HCl buffer (hereafter referred to as “0 sample”), as well as after each homogenization cycle (up to five cycles), samples were taken. Samples were centrifuged (14,000 rpm, 4 °C, 15 min), and the supernatants were used for further analyses.

### Analytical methods

#### HPLC-measurements

##### Data acquisition

UV chromatographic data were acquired using the PATfinder™ analytical device (BIAseparations, Ajdovščina, Slovenia) comprising of an autosampler (Optimas), a pump (Azura P 6.1L), a UV detector (Azura MWD 2.1L) and a monolithic CIMac QA column (0.1 mL). UV chromatographic data at 280 nm were recorded at 5 Hz to monitor the total protein content. According to our previous study, where we successfully used this setup for monitoring cell integrity during bioreactor cultivations [25], the monolithic column was equilibrated with 10 column volumes (CV) of loading buffer (50 mM TRIS–HCl buffer, pH 8.0), followed by 50 µL of sample injection and a post injection wash of 10 CV loading buffer. The bound proteins and nucleic acids were eluted with 100% elution buffer (50 mM Tris–HCl, 1 M NaCl, pH 8) for 10 CV before the column was stripped with 1 M NaOH/2 M NaCl for 10 CV to avoid carry over. The flow velocity was maintained at 280 cm/h throughout the whole HPLC run resulting in a total analysis time of 5 min per sample.

##### Automated data processing

The total areas under the curve (AUC) for the flowthrough (FT) and elution (EL) peaks were used to follow the relative increase of the protein content in the supernatants after homogenization. The individual chromatograms from samples at different steps were automatically imported using MATLAB (Mathworks, US; vR2016a). A reference spectrum was generated based on the arithmetic mean or average of all imported UV chromatograms at 280 nm, since this is a prerequisite for peak alignment and generation of chromatogram fingerprints. Peak alignment was done in MATLAB using icoshift [29]. Thereon, automated integration of the peaks in the region of interest, namely FT and EL, was done using the Trapz function in MATLAB. Finally, the AUC was used to calculate the relative recovery of proteins. The relative increase of total protein content was calculated using Eq. 2,

2 $${\text{Relative protein recovery}} = \frac{{AUC_{i} - AUC _{Start} }}{{AUC_{End} - AUC_{Start} }}*100,$$

where AUC_i_ is the total AUC of sample I, AUC_Start_ the total AUC of the first sample and AUC_End_ is the total AUC of the last sample.

#### Reference analytics

We used several established state-of-the-art reference analytics to evaluate cell disruption efficiency, and thus our methodology based on HPLC and automated data analysis.

##### Protein concentration

We determined the total protein content by the Bradford Coomassie Blue assay (Sigma-Aldrich, Vienna, Austria) and used bovine serum albumin as standard. To stay in the linear range of the UV detector (Genesys 20, Thermo Scientific, Waltham, MA, USA) from 0.1 to 0.8 absorption units, samples were diluted with water, when necessary.

##### Colony forming Units (CfUs)

To judge viable cell reduction, we determined the Colony forming Units (CfUs). For that purpose, cell suspensions were diluted with sterile 0.9% (w/v) NaCl solution in 1:10 steps ranging from 10^0^ to 10^−16^. 100 µL of each dilution were plated out on selective Agar plates (10 g/L tryptone, 5 g/L yeast extract, 10 g/L NaCl, 15 g/L plate count agar, pH 7.5) containing 50 µg/mL kanamycin. After incubation at 35 °C for 48 h, formed colonies were counted.

##### Flow cytometric (FC) measurements

Flow cytometric (FC) analysis was carried out according to Langemann et al. [30]. Samples were diluted with particle-free, sterile 0.9% (w/v) NaCl solution, to avoid oversaturation of the detector (CyFlow^®^ Cube 8 flow cytometer; Partec, Münster, Germany). RH414 (abs./em. 532/760 nm, directed to plasma membranes) and DiBAC4(3) (abs./em. 493/516 nm, membrane potential-sensitive dye) dyes were mixed with the samples shortly before analysis. Using both dyes concomitantly, quantification of all cells (RH414), dead cells [DiBAC4(3)] and viable cells [signal of RH414 minus signal of DiBAC4(3)] was possible. Measurements were recorded by the CyView Cube 15 software and evaluated with FCS Express V4 (DeNovo Software; Los Angeles, CA, USA).

##### Dielectric spectroscopy (DS)

Dielectric spectroscopy (DS) is usually used to follow viable cell concentrations (VCC) in upstream processes [31]. In this study, DS was used to track viable cell reduction, as an additional method. Low radio frequencies lead to a polarization of cells due to charge separation effects. At high frequencies, no polarization of cells can be observed. Here mainly background, such as water dipoles, is measured [32]. In this study, two frequencies, 1 MHz for viable bacterial cells and 10 MHz for non-cellular background, were used during dielectric measurements in standard dual-frequency measuring mode [33]. The difference between those two frequencies led to the measured parameter, namely delta capacitance. Measurements were performed with a FOGALE nanotech probe (HAMILTON Bonaduz AG, Bonaduz, Switzerland). Its signal was logged using the Evobox software (HAMILTON Bonaduz AG, Bonaduz, Switzerland). Untreated and disrupted cell suspensions were measured for at least 3 min. The mean value of this signal was used for the calculation of percental signal reduction corresponding to the decrease in cell viability.

### Experimental design

The experiments in this study were divided into three work packages (WPs), namely WP1. State-of-the-art methods compared to HPLC combined with automated data analysis, WP2. Effect of freezing on cell disruption efficiency, and WP3. Development of a cell disruption process by a design of experiment approach. More details about the three WPs are given in Table 3.

**Table 3:** Overview of the three experimental work packages (WPs)

WP	Strategy	Analytics	Goal
1	DCW: 10 g/L Pressure: 1500 bar Cycles: 0–5	Bradford = state-of-the-art Flow cytometry = state-of-the-art CfUs = state-of-the-art Dielectric spectroscopy = additional method HPLC and automated data analysis = novel method	Evaluation of applicability and accuracy of our method
2	DCW: 10 g/L Pressure: 1500 bar Cycles: 0–5	Bradford HPLC and automated data analysis	Analyzing potential effects of freezing on cell disruption efficiency
3	DCW: 10–100 g/L Pressure: 500–1500 bar Cycles: 0–3	Bradford HPLC and automated data analysis	Evaluation the effect of BM concentration, pressure and cycles on cell disruption

#### WP1. State-of-the-art methods compared to HPLC combined with automated data analysis

The goal of this WP was to evaluate the applicability and accuracy of our method of HPLC and automated data analysis, which we successfully used in upstream processing [25], to analyze cell disruption efficiency. Thus, we wanted to demonstrate the applicability of our tool across unit operations. In WP1, we homogenized resuspended *E. coli* BM at 1500 bar for five cycles and analyzed the disruption efficiency by five different methods (Table 3).

#### WP2. Effect of freezing on cell disruption efficiency

Different factors, like time management and occupancy of equipment, can cause the necessity of holding steps in a production process. Freezing the BM after harvesting presents such a typical holding step (Fig. 1). In WP2, we analyzed potential effects of freezing on cell disruption efficiency. Thus, resuspended BM was either homogenized directly or frozen at −20 °C for at least 24 h, followed by thawing at 4 °C and high-pressure homogenization at 1500 bar for five cycles (Table 3).

#### WP3. Development of a cell disruption process by a design of experiment approach

**Fig. 2:**
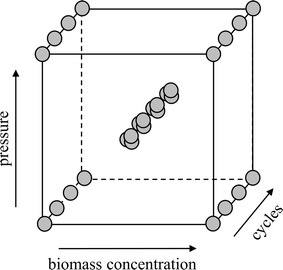
Design space and respective experiments of the full factorial screening study performed in WP3. Homogenization pressure (500, 1000 and 1500 bar) and cell density (10, 55 and 100 g DCW/L) were used as quantitative factors. The number of homogenization cycles (0, 1, 2 and 3) was used as a quantitative multilevel factor

The goal of WP3 was to evaluate the effect of the three factors “biomass concentration (10–100 g DCW/L)”, “number of cycles (0–3)” and “homogenization pressure (500–1500 bar)” on cell disruption efficiency. For that purpose, we designed a full factorial screening study using the software MODDE10 (Umetrics, Umeå, Sweden). The respective design space is shown in Fig. 2.

## Results and discussion

### WP1. State-of-the-art methods compared to HPLC combined with automated data analysis

**Fig. 3:**
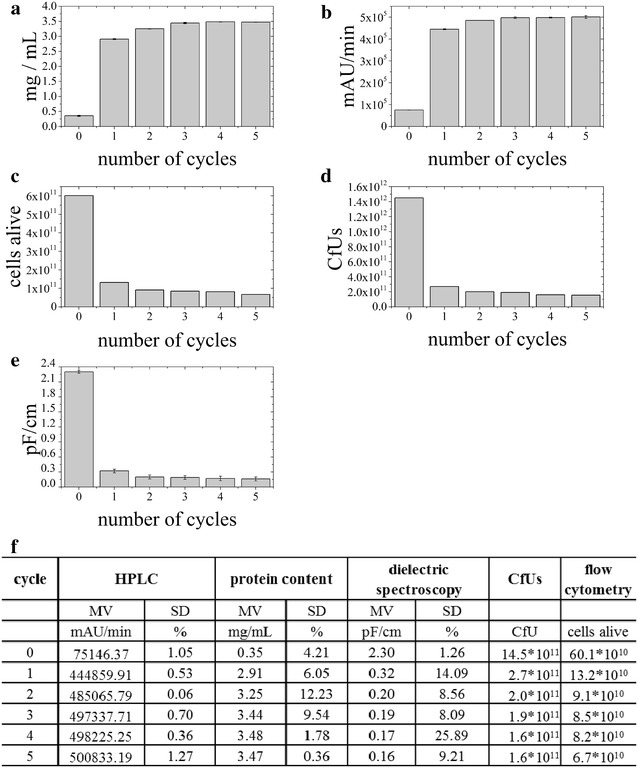
Raw data obtained from different analytical methods to evaluate cell disruption efficiency. **a** Total released protein (mg/mL) determined by Bradford, **b** area under the curve (AUC) measured with HPLC, **c** decrease of viable cells determined by flow cytometric measurements, **d** decrease of Colony forming Units (CfUs) and **e** reduction of the dielectric spectroscopy signal. **f** Summary of data: actual values, as mean value (MV) with standard deviation (SD), if analytics was performed in triplicates

In WP1, we compared different methods to evaluate cell disruption efficiency. In Fig. 3 the respective raw data are shown.

To be able to easily compare the different analytical methods, the respective raw data were normalized and are shown relatively in % in Table 4.

**Table 4:** Comparison of normalized data from five different analytical methods to evaluate cell disruption efficiency

Cycle	Total protein content [%]		Signal reduction [%]	Viable cells [%]	
	Bradford	HPLC	DS	CfUs	FC
0	10.2	15.0	100.0	100.0	100.0
1	83.8	88.8	13.9	18.3	21.9
2	93.7	96.9	8.7	13.8	15.1
3	99.2	99.3	8.3	13.1	14.1
4	100.3	99.5	7.4	11.0	13.6
5	100.0	100.0	7.1	10.7	11.1

The total protein content after five cycles was considered to be 100%. Based on this assumption %-values for the other cycles were calculated

As shown in Table 4, all five analytical methods gave comparable results. After the first homogenization cycle at 1500 bar, around 80–90% of the cells were disrupted. The second homogenization cycle reduced the amount of intact cells by another 5–10%, whereas following homogenization cycles only resulted in minor additional cell disruption. By this comparative analysis, we were able to prove that our method of using HPLC followed by automated data analysis describes a valid tool, not only to follow cell integrity in the USP, but also to monitor cell disruption efficiency in the DSP. Compared to the state-of-the-art methods, our method is automated, only takes 5 min per sample and can be implemented at-line.

### WP2. Effect of sample freezing on cell disruption efficiency

**Fig. 4:**
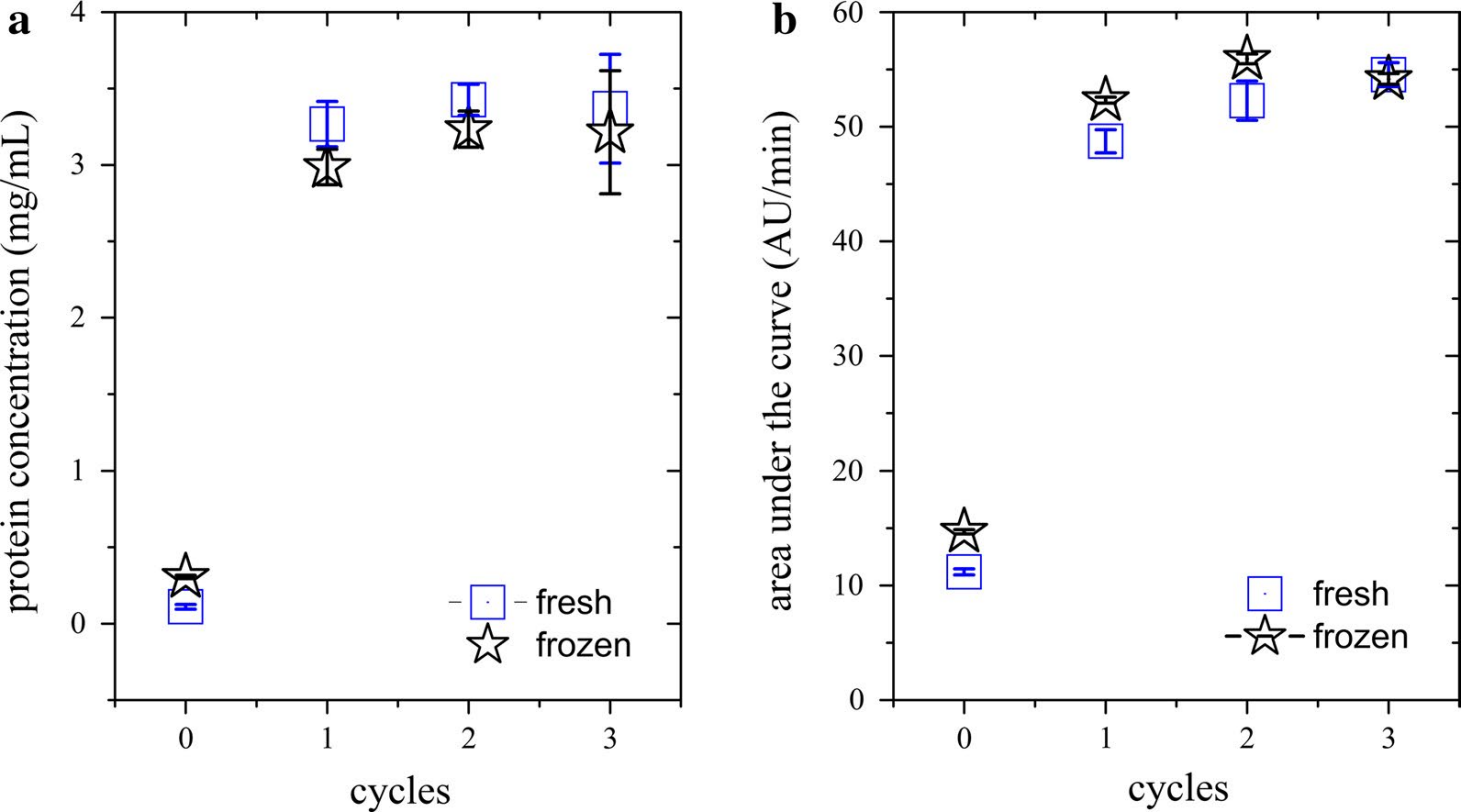
Disruption efficiency monitored by (**a**), Bradford measurements or (**b**), HPLC followed by automated data analysis. *Stars*, frozen biomass; *squares*, fresh biomass

Freezing of BM after harvest describes a common hold step in a recombinant protein production process (indicated in red in Fig. 1). In WP2, we investigated whether freezing affects the subsequent cell disruption efficiency. For that purpose, we homogenized BM, either directly after harvest or thawed at 4 °C after previous freezing at −20 °C, for three cycles at 1500 bar. In this WP, we only investigated three homogenization cycles, since in WP1 we had found that additional cycles did not significantly contribute to cell disruption (Table 4). We analyzed the respective supernatants by Bradford measurements and HPLC followed by automated data analysis. As shown in Fig. 4, both analytical methods reveal the same outcome. The first homogenization cycle reduced the amount of intact cells by a factor of around 90%, no matter if the BM had been frozen before or not. The following homogenization cycles only slightly increased the amount of disrupted cells in both cases. However, when BM had been frozen before, around twice the amount of protein was measured already before homogenization indicating that cells had already lysed by freezing/thawing (Fig. 4). Nonetheless, we concluded that freezing the BM after harvest is an acceptable hold step in a recombinant protein production process, as it does not affect the cell disruption efficiency by high-pressure homogenization. However, potential effects of freezing on the recombinant product have to be evaluated on a case-by-case basis as those effects are product-specific.

### WP3. Development of a cell disruption process by a design of experiment approach

**Fig. 5:**
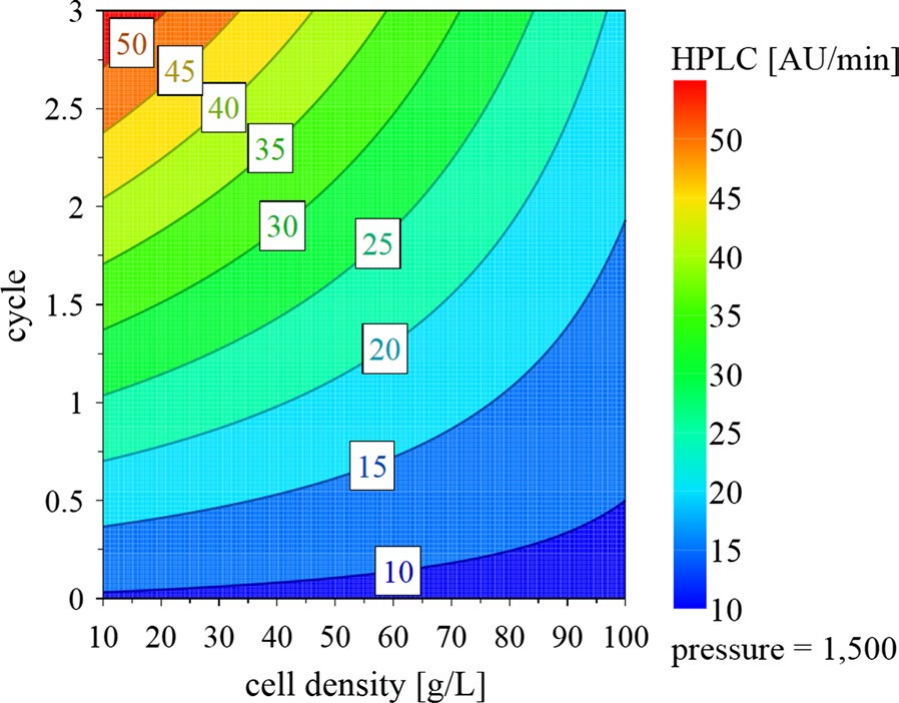
Response contour plot of protein release during high-pressure homogenization. Goodness of fit (R^2^) = 0.892; goodness of prediction (Q^2^) = 0.813

According to literature, no difference in homogenization efficiency is seen if BM concentrations are kept below 12.5 g DCW/L [18]. Also, protein release was found to be insufficient at a pressure below 500 bar [18]. Hence, for the DoE screening design we investigated BM concentrations from 10 to 100 g DCW/L, as well as pressure settings from 500 to 1500 bar. Furthermore, we investigate the number of homogenization cycles between 0 and 3. The respective design space is shown in Fig. 2. We used Bradford measurements as well as HPLC followed by automated data analysis to monitor cell disruption efficiency under the different conditions. Both analytical methods showed no significant difference, underlining the validity of the novel method we present here. In Fig. 5 a contour plot showing the results of the DoE screening design evaluated by HPLC and automated data analysis is shown. To be able to directly compare the effect of the different factors on cell disruption efficiency, the AUC signals were normalized to the biomass before multivariate data evaluation.

As shown in Fig. 5, cell disruption efficiency increased with an increasing number of homogenization cycles at 1500 bar and low biomass concentrations. With respect to biomass concentration and homogenization efficiency, literature is quite inconsistent, as some studies report no effect [22], while others do [34, 35]. In our study, we observed higher disruption efficiency for samples with lower biomass concentration. The homogenization pressure had no significant impact on cell disruption efficiency in the tested ranges (*p* value = 0.87). We did not investigate the effect of these settings on the recombinant product, since the impact of pressure and number of homogenization cycles is certainly product-dependent. However, we provide an automated platform methodology to evaluate cell disruption efficiency, which will enable fast development of a cell disruption strategy tailored to specific products allowing both high cell disruption efficiency and prevention of product loss.

## Conclusions

Cell disruption is a key unit operation to access recombinant intracellular protein from *E. coli*. Thus, monitoring tools are needed to evaluate cell disruption strategies and resulting cell disruption efficiency. However, current state-of-the-art methods are time-delayed, slowing down process development, and require manual intervention, making them error-prone. In the present study, we applied a methodology comprising HPLC and automated data analysis, which we recently developed to monitor upstream processes, to evaluate cell disruption efficiency of high-pressure homogenization. Our findings can be summarized as:

- HPLC followed by automated data analysis outcompetes current state-of-the-art methods to monitor cell disruption efficiency, as it is faster and does not require manual intervention.
- Freezing of BM prior to high-pressure homogenization has no impact on cell disruption efficiency.
- The biomass concentration and the number of homogenization cycles affect cell disruption efficiency, whereas the pressure can be varied between 500 and 1500 bar without significant impact.

We are convinced that our methodology will be the golden standard to evaluate cell disruption processes in the future as it can be implemented at-line, gives results within minutes after sampling and does not need manual intervention. This tool does not only allow the fast development of cell disruption strategies specifically tailored to protect the product, but actually describes a useful tool applicable across unit operations.

## Abbreviations

AU: absorption units
AUC: area under the curve
BM: biomass
CfUs: Colony forming Units
CV: column volume
DCW: dry cell weight
DoE: design of experiment
DS: dielectric spectroscopy
DSP: downstream processing
EL: elution
FC: flow cytometry
FT: flow through
IB: inclusion body
IPTG: isopropyl β-d-1-thiogalactopyranoside
MV: mean value
OD_600_: optical density measured at 600 nm
SB: super broth
SB-Kan: super broth supplemented with 50 µg/mL kanamycin
scFv: single chain fragment variable
SD: standard deviation
SF: shake flask
USP: upstream processing
WPs: work packages
